# Delivery unit volume and neonatal mortality — A nationwide register study in Finland from 2008 to 2023

**DOI:** 10.1007/s00431-025-06133-5

**Published:** 2025-04-12

**Authors:** Ilari Kuitunen

**Affiliations:** 1 University of Eastern Finland, Kuopio, Finland; 2 Department of Pediatrics, Kuopio University Hospital, Puijonlaaksontie 2, 70211 Kuopio, Finland

**Keywords:** Neonatal mortality, Cohort study, Register study

## Abstract

This nationwide study analyzed neonatal mortality in Finland from 2008 to 2023, focusing on the impact of annual delivery volumes and their changes across hospitals. Data from 819,465 liveborn neonates, including 46,906 preterm births, were analyzed using the Finnish Medical Birth Register. While neonatal mortality rates showed an overall decline—driven by improved preterm survival—term neonatal mortality remained stable. The lowest neonatal mortality rate was recorded in 2020–2021 at 0.88 per 1000 live births, with preterm mortality at 9.85 per 1000. No clear associations were identified between delivery volume, volume changes, and neonatal outcomes in tertiary or secondary units. Despite declining birth volumes and increasing small-volume hospitals, neonatal outcomes remained consistent.

*Conclusion*: These findings challenge arguments for further centralization of births based on volume thresholds in Finland. Continuous monitoring of neonatal outcomes is essential amid decreasing fertility rates and evolving healthcare systems to sustain Finland’s excellence in neonatal care.

**Supplementary Information:**

The online version contains supplementary material available at 10.1007/s00431-025-06133-5.

## Introduction

Finland has been traditionally ranked among the countries with the lowest neonatal and infant mortality rates globally [1, 2]. According to the most recent European collaboration study, Finland recorded the lowest mortality rates [3]. In Finland, births of very preterm neonates and cases involving certain congenital anomalies have been centralized in tertiary-level university hospitals, which has improved the outcomes [4].

However, Finland has recently introduced legislation requiring special exemptions for hospitals with an annual delivery volume of fewer than 1000 cases. Additionally, decreasing government funding has prompted discussions about further centralization of operations, including reducing the number of birth hospitals. Concerns have been raised that this could lead to an increase in both planned and unplanned deliveries outside hospital settings [5]. The debates surrounding these changes have primarily focused on financial and political considerations.

The most recent Finnish report analyzing neonatal outcomes across hospitals was published in 2011 [6]. It concluded that there was no need for further centralization of preterm births occurring after 31 + 6 weeks of gestation. However, since then, Finland’s fertility rate has declined rapidly, leading to a decrease in the average number of deliveries per unit [7]. This study aims to analyze neonatal mortality in relation to annual delivery volume and changes in delivery volume across hospitals.

## Methods

This nationwide register-based study contains information on all pregnancies ending in delivery in Finland from 2008 to 2023. The data was obtained from the open-access database of the Finnish Medical Birth Register. The medical birth register contains information on all pregnancies ending in delivery after gestational week 21 + 6 or fetal weight more than 499 g. For this study, all deliveries (including singletons and multiples) occurring in currently operating delivery hospitals were included. Still births were excluded. Deliveries were included from five tertiary level units and 18 secondary level units. Births of very preterm neonates (gestational weeks less than 32 + 0) and some congenital anomalies have been centralized into tertiary level units in Finland. Thus, in the comparisons, we will focus on within comparisons, and not compare tertiary units to secondary units. Preterm birth was classified as birth occurring before gestational week 37 + 0. Incidences of early neonatal mortality (death during the first 7 days of life) per 1000 liveborn neonates were calculated with 95% confidence intervals (CI). In the calculations, we used the annual means of deliveries from 2008 to 2023. Furthermore, we compared the neonatal mortality to the mean annual delivery volume change from 2008–2009 to 2022–2023 to analyze the association with the decreasing unit volume. A linear mixed regression model with fixed effects for hospital was used to analyze the association between hospital volume and both relative and absolute change in annual delivery volumes to neonatal mortality. Analyses were made in SPSS version 29.0.

As this study was based on open-access data, no research permissions or participant contents were required.

## Results

**Fig. 1:**
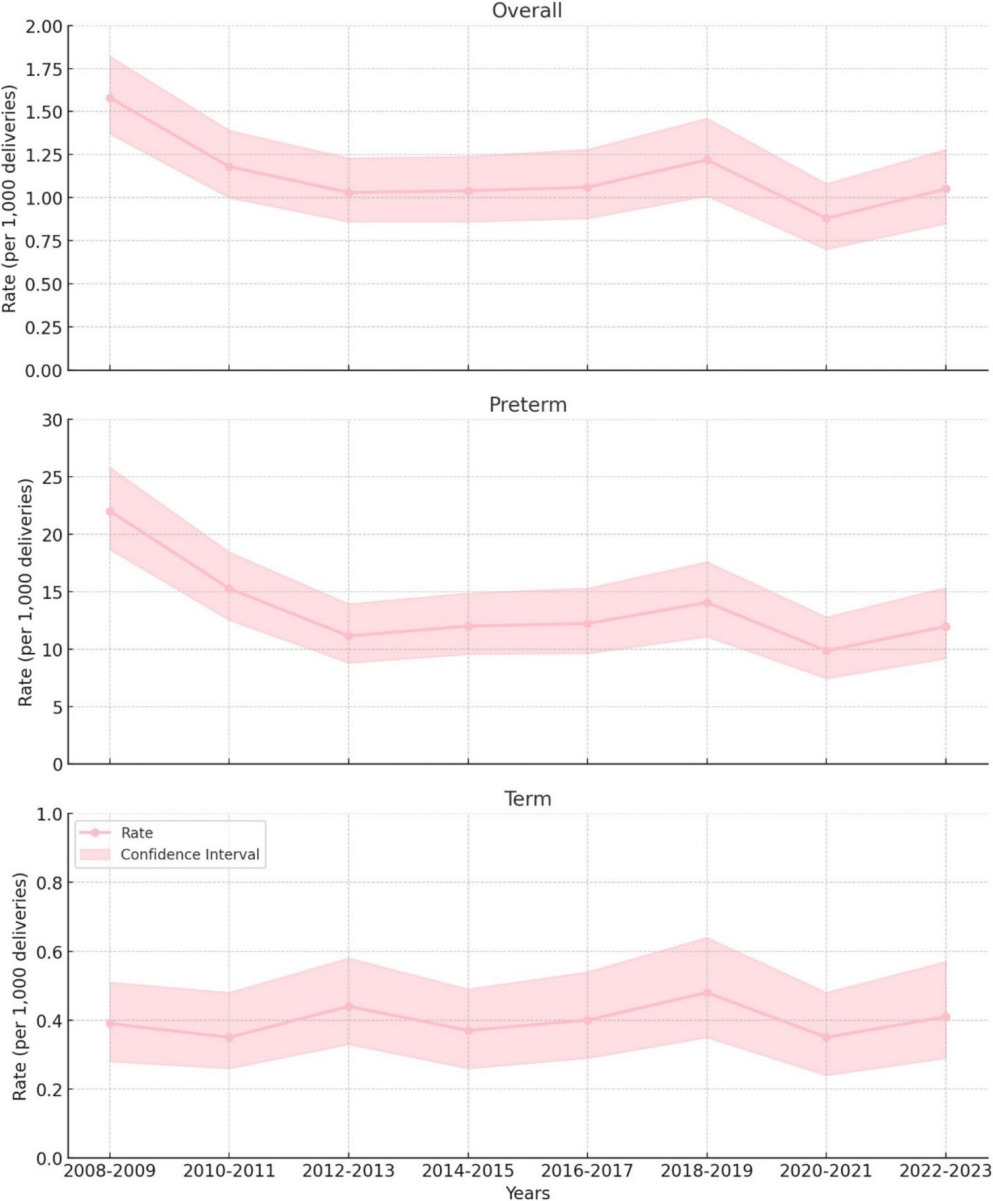
The neonatal mortality rate in Finland per 1000 deliveries with 95% confidence intervals (CI) from 2008 to 2023

A total of 821,835 births occurred during the study period, of which 819,465 neonates were born alive and included for this study. Of these, 402,393 (49.1%) occurred in tertiary level hospitals. Overall, a total of 46,906 (5.7%) live born preterm neonates were included, and of these, 30,035 (64.0%) were delivered in tertiary level units. The overall neonatal mortality rate has shown a decreasing trend and remained in low levels, and this decrease has been due to improvement in the preterm survival rate, as the term mortality rate has remained practically unchanged (Fig. 1). The lowest reported mortality rates were in 2020–2021, as the overall neonatal mortality rate was 0.88 (CI: 0.70–1.08) per 1000. For preterm neonates, the mortality rate was 9.85 (CI: 7.45–12.78) per 1000 in 2020–2021. During the study period, the lowest mortality rate for term neonates was 0.35 per 1000 in 2010–2011, and 2020–2021 (Fig. 1).

**Fig. 2:**
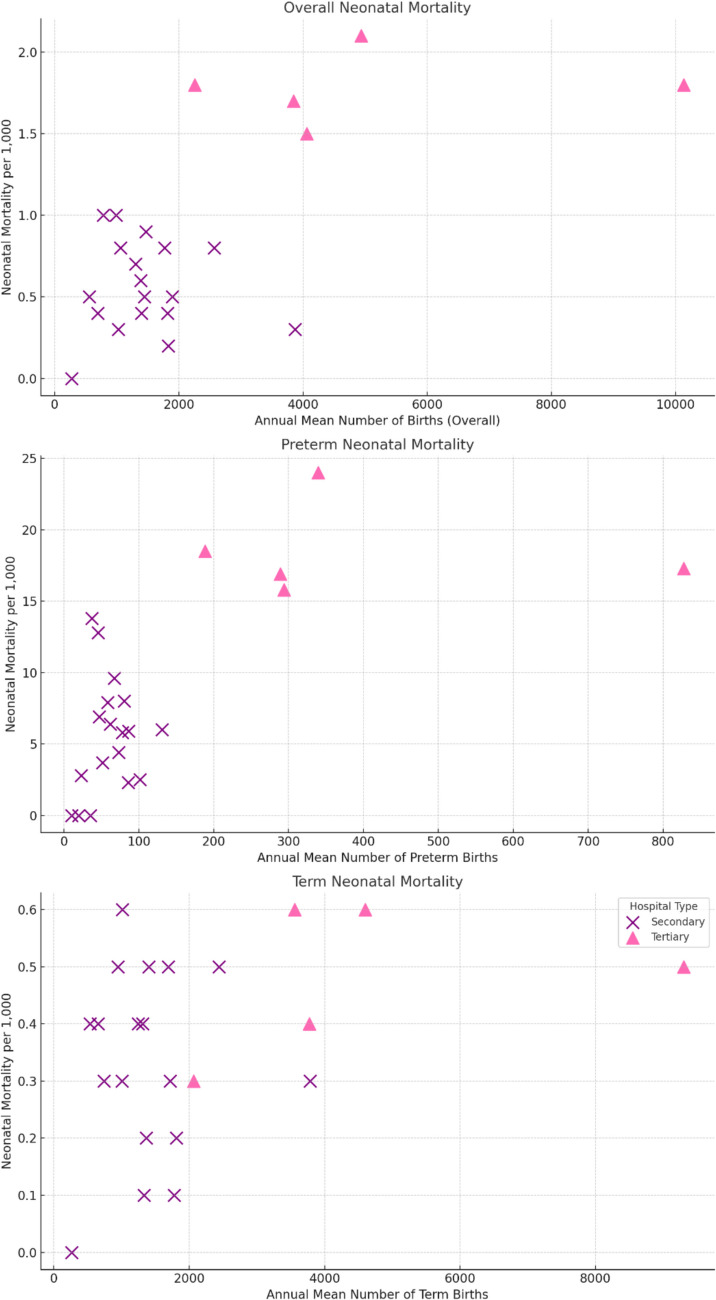
Neonatal mortality rate per 1000 deliveries based on the mean annual delivery unit volume. Analysis stratified into overall, preterm, and term births. Tertiary level hospitals and secondary level units are presented separately in the graphs

By examining the trends between the hospital’s annual delivery volume, no clear association was seen in the volume and neonatal outcomes overall, or preterm neonates either in secondary or tertiary level units (Fig. 2; Table S1). Similarly, there was no trend between the relative and absolute change in the annual delivery unit volume and neonatal outcomes (Fig. S1; Table S2).

## Discussion

These results showed that Finland has managed to decrease preterm neonatal mortality rates and keep the term mortality rates in low levels. Despite the decreased annual birth volumes and increase in the number of small volume birthing hospitals, the neonatal mortality outcomes have not worsened. These findings do not support the closure of current delivery units, as there were no clear trends based on the overall delivery unit volume, or with the delivery unit volume change. These findings indicate that the current centralization of high-risk cases have been effective. The majority of the previous literature has focused on the centralization of very preterm births or very low-birth weight neonates, and these studies have found improved survival and less morbidity in larger units [8–10]. The literature focusing of moderate and late preterm outcomes is more limited. One systematic review analyzed low risk births and reported an association between delivery unit volume and improved neonatal outcomes [11]. However, meta-analysis was not conducted due to major heterogeneity in the study settings and definitions. The studies had used various thresholds from 500 to 1000 in the low volume unit definition. In Finland, the threshold has been 1000 deliveries per year, and currently seven of the 23 birth hospitals in Finland have less deliveries.

A clear limitation is that this study is based on the materials available in the open-access database, and thus the reporting is limited and controlling for confounding factors was not possible. However, this may be seen also as a strength as this enables the prompt reporting, as currently due to legislation changes the waiting times to individual level datasets have been prolonged up to 12 months in Finland [12]. Furthermore, the coverage and the quality of the Finnish birth register data has been excellent [13].

These findings provide important material for the discussion on the centralization of births, and due to already ongoing centralization of high-risk pregnancies to tertiary units, the current findings of this study show no clear association between the delivery unit volume and neonatal mortality rates in Finland. These current desires to re-organize delivery units come from funding and politics in Finland. Due to changes in the healthcare organizations and continuous decrease in fertility rate in Finland, the continuous monitoring of neonatal outcomes is needed to maintain the current excellence in the care of neonates.

## Supplementary Information

Below is the link to the electronic supplementary material.

Supplementary file1 Hospital specific statistics and annual neonatal mortality rates with 95% confidence intervals (CI). Additional analyses and tables. (DOCX 189 kb)

## Data Availability

All data available from the open-access database of the Finnish Medical Birth Register: https://thl.fi/en/statistics-and-data/statistics-by-topic/sexual-and-reproductive-health/parturients-deliveries-and-births/perinatal-statistics-parturients-delivers-and-newborns.
