# Supplementary material for: Delivery unit volume and neonatal mortality — A nationwide register study in Finland from 2008 to 2023
Source: Eur J Pediatr. 2025 Apr 12;184(5):293. doi: 10.1007/s00431-025-06133-5 (PMC11993435; doi:10.1007/s00431-025-06133-5)
Supplement: Supplementary file 1 — Supplementary file1 Hospital specific statistics and annual neonatal mortality rates with 95% confidence intervals (CI). Additional analyses and tables. (DOCX 189 kb) [file 431_2025_6133_MOESM1_ESM.docx]

**Supplementary material**

Figure S1: Neonatal mortality and annual % and absolute change in delivery units in Finland from 2008 to 2023.


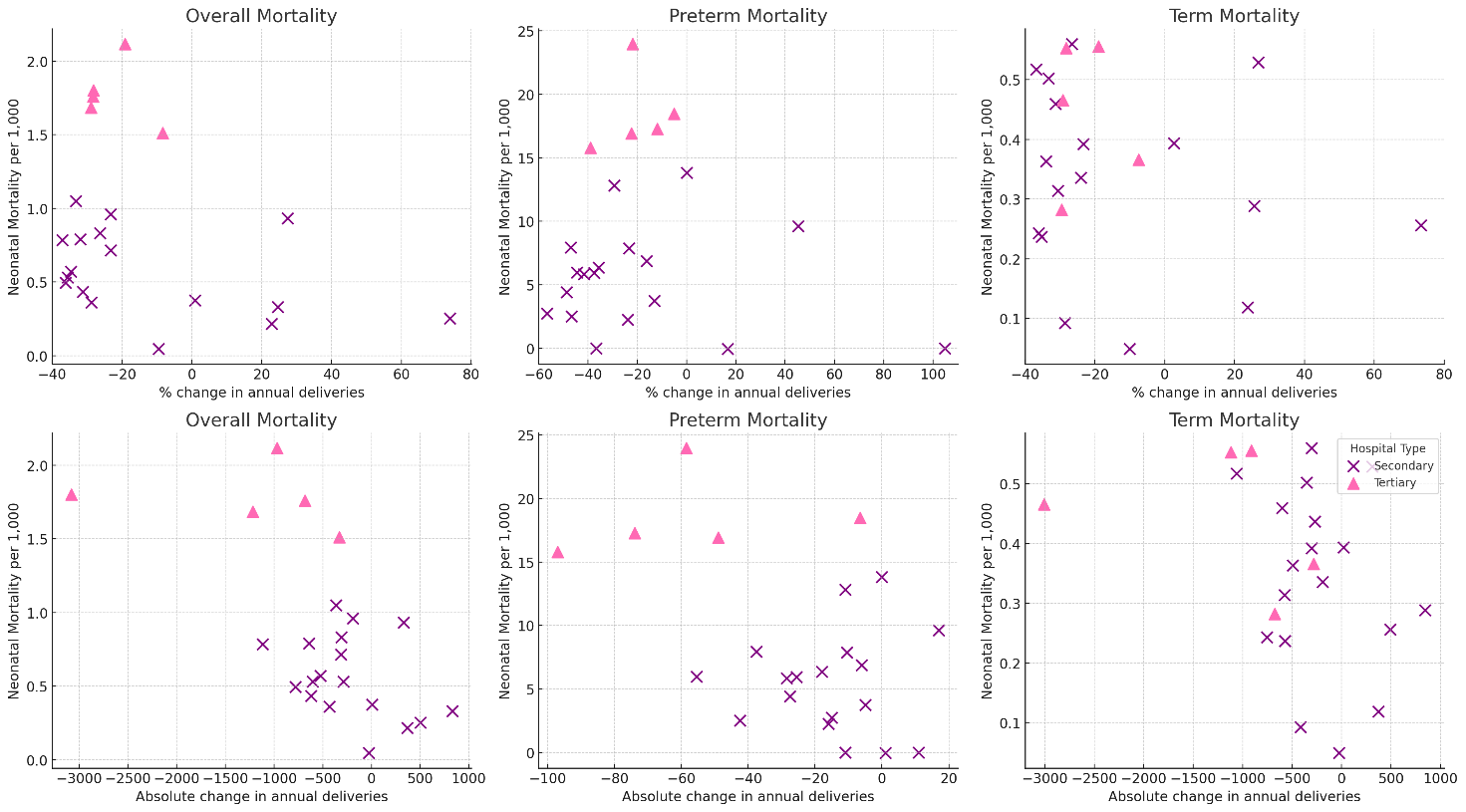


Table S1: Mixed linear regression fixed effect estimates for the analyses presented in Figure 2 on the association between birth hospital annual volume and neonatal mortality.

| **Hospital Type** | **Outcome** | **Predictor** | **Estimate** | **95% CI Lower** | **95% CI Upper** | **p-value** |
| --- | --- | --- | --- | --- | --- | --- |
| Secondary | Overall | Annual Births Overall | -0,00004 | -0,00019 | 0,00010 | 0,53123 |
| Secondary | Preterm | Annual Births Preterm | 0,01604 | -0,05032 | 0,08240 | 0,61667 |
| Secondary | Term | Annual Births Term | 0,00000 | -0,00008 | 0,00009 | 0,94547 |
| Tertiary | Overall | Annual Births Overall | 0,00001 | -0,00012 | 0,00014 | 0,81362 |
| Tertiary | Preterm | Annual Births Preterm | -0,00151 | -0,02484 | 0,02183 | 0,85045 |
| Tertiary | Term | Annual Births Term | 0,00002 | -0,00007 | 0,00010 | 0,57338 |

Table S2: Mixed linear regression fixed effect estimates for the analyses presented in Figure S1 on the association between relative and absolute change annual delivery volume and neonatal mortality.

| **Hospital Type** | **Outcome** | **Predictor** | **Estimate** | **95% CI Lower** | **95% CI Upper** | **p-value** |
| --- | --- | --- | --- | --- | --- | --- |
| Secondary | Overall | % Change Overall | -0,0034 | -0,0079 | 0,0010 | 0,1187 |
| Secondary | Preterm | % Change Preterm | -0,0120 | -0,0651 | 0,0410 | 0,6371 |
| Secondary | Term | % Change Term | -0,0010 | -0,0036 | 0,0015 | 0,3928 |
| Secondary | Overall | Absolute Change Overall | -0,0002 | -0,0005 | 0,0001 | 0,1409 |
| Secondary | Preterm | Absolute Change Preterm | 0,0088 | -0,1072 | 0,1248 | 0,8745 |
| Secondary | Term | Absolute Change Term | -0,0001 | -0,0002 | 0,0001 | 0,3475 |
| Tertiary | Overall | % Change Overall | -0,0064 | -0,0503 | 0,0375 | 0,6736 |
| Tertiary | Preterm | % Change Preterm | 0,0530 | -0,3978 | 0,5038 | 0,7331 |
| Tertiary | Term | % Change Term | -0,0011 | -0,0240 | 0,0219 | 0,8902 |
| Tertiary | Overall | Absolute Change Overall | 0,0000 | -0,0004 | 0,0003 | 0,7265 |
| Tertiary | Preterm | Absolute Change Preterm | 0,0239 | -0,1466 | 0,1945 | 0,6855 |
| Tertiary | Term | Absolute Change Term | 0,0000 | -0,0002 | 0,0002 | 0,6165 |

Hospital specific neonatal mortalirty per 1,000 liveborn neonates.

| **Hospital** | **Neonatal mortality overall** | **Annual mean of births** | **Neonatal mortality term** | **Annual mean of term births** | **Neonatal mortality preterm** | **Annual mean of preterm births** |
| --- | --- | --- | --- | --- | --- | --- |
| **Tertiary 1** | 1,8 | 10130,8 | 0,5 | 9303,3 | 17,3 | 827,5 |
| **Tertiary 2** | 1,8 | 2254,9 | 0,3 | 2066,6 | 18,5 | 188,4 |
| **Tertiary 3** | 1,7 | 3848,9 | 0,6 | 3555,0 | 15,8 | 293,9 |
| **Tertiary 4** | 2,1 | 4938,4 | 0,6 | 4599,1 | 24,0 | 339,3 |
| **Tertiary 5** | 1,5 | 4062,8 | 0,4 | 3774,0 | 16,9 | 288,8 |
| **Secondary 1** | 1,0 | 990,9 | 0,5 | 945,0 | 12,8 | 45,9 |
| **Secondary 2** | 0,5 | 1895,9 | 0,2 | 1809,5 | 5,9 | 86,4 |
| **Secondary 3** | 0,4 | 691,6 | 0,4 | 656,3 | 0,0 | 35,3 |
| **Secondary 4** | 0,5 | 1443,5 | 0,2 | 1365,5 | 5,8 | 78,0 |
| **Secondary 5** | 0,9 | 1471,2 | 0,5 | 1404,0 | 9,6 | 67,2 |
| **Secondary 6** | 0,8 | 2571,1 | 0,5 | 2440,4 | 6,0 | 130,8 |
| **Secondary 7** | 0,4 | 1396,3 | 0,1 | 1334,6 | 6,4 | 61,7 |
| **Secondary 8** | 0,8 | 1058,2 | 0,6 | 1011,1 | 6,9 | 47,1 |
| **Secondary 9** | 0,5 | 556,5 | 0,4 | 533,4 | 2,8 | 23,1 |
| **Secondary 10** | 1,0 | 780,9 | 0,3 | 743,7 | 13,8 | 37,2 |
| **Secondary 11** | 0,6 | 1385,5 | 0,4 | 1312,5 | 4,4 | 73,0 |
| **Secondary 12** | 0,8 | 1771,6 | 0,5 | 1691,2 | 8,0 | 80,4 |
| **Secondary 13** | 0,4 | 1819,3 | 0,3 | 1717,8 | 2,5 | 101,5 |
| **Secondary 14** | 0,7 | 1301,9 | 0,4 | 1243,8 | 7,9 | 58,2 |
| **Secondary 15** | 0,0 | 270,8 | 0,0 | 260,8 | 0,0 | 10,1 |
| **Secondary 16** | 0,2 | 1827,6 | 0,1 | 1775,9 | 3,7 | 51,7 |
| **Secondary 17** | 0,3 | 1026,3 | 0,3 | 1006,8 | 0,0 | 19,5 |
| **Secondary 18** | 0,3 | 3869,9 | 0,3 | 3783,9 | 2,3 | 86,0 |

Hospital specific absolute and % changes in annual delivery volume.

|  | % change overall | Absolute change overall | % change preterm | Absolute change preterm | % change full term | Absolute change full term |
| --- | --- | --- | --- | --- | --- | --- |
| **Tertiary 1** | -28 % | -3081,5 | -12 % | -74 | -29 % | -3007,5 |
| **Tertiary 2** | -28 % | -682 | -5 % | -6,5 | -30 % | -675,5 |
| **Tertiary 3** | -29 % | -1216,5 | -39 % | -97 | -28 % | -1119,5 |
| **Tertiary 4** | -19 % | -970,5 | -22 % | -58,5 | -19 % | -912 |
| **Tertiary 5** | -8 % | -331 | -22 % | -49 | -8 % | -282 |
| **Seconadry 1** | 25 % | 829,5 | -24 % | -16 | 26 % | 845,5 |
| **Secondary 2** | -33 % | -362,5 | -29 % | -11 | -33 % | -351,5 |
| **Secondary 3** | -36 % | -780 | -38 % | -25,5 | -36 % | -754,5 |
| **Secondary 4** | 1 % | 7 | -37 % | -11 | 3 % | 18 |
| **Secondary 5** | -36 % | -602,5 | -42 % | -28,5 | -35 % | -574 |
| **Secondary 6** | 27 % | 328 | 45 % | 17 | 27 % | 311 |
| **Secondary 7** | -37 % | -1116,5 | -44 % | -55,5 | -37 % | -1061 |
| **Secondary 8** | -29 % | -432 | -36 % | -18 | -29 % | -414 |
| **Secondary 9** | -26 % | -308 | -16 % | -6 | -27 % | -302 |
| **Secondary 10** | -42 % | -286,5 | -57 % | -15 | -42 % | -271,5 |
| **Secondary 11** | -23 % | -192 | 0 % | 0 | -24 % | -192 |
| **Secondary 12** | -35 % | -522,5 | -49 % | -27,5 | -34 % | -495 |
| **Secondary 13** | -32 % | -640 | -47 % | -37,5 | -31 % | -602,5 |
| **Secondary 14** | -31 % | -619 | -47 % | -42,5 | -31 % | -576,5 |
| **Secondary 15** | -23 % | -314 | -23 % | -10,5 | -23 % | -303,5 |
| **Secondary 16** | -9 % | -25,5 | 17 % | 1 | -10 % | -26,5 |
| **Secondary 17** | 23 % | 367,5 | -13 % | -5 | 24 % | 372,5 |
| **Secondary 18** | 74 % | 504 | 105 % | 11 | 73 % | 493 |

Annual neonatal mortality per 1,000 liveborn neonates with 95% confidence intervals.

|  | 2008–2009 | 2010–2011 | 2012–2023 | 2014–2015 | 2016–2017 | 2018–2019 | 2020–2021 | 2022–2023 |
| --- | --- | --- | --- | --- | --- | --- | --- | --- |
| OVERALL |  |  |  |  |  |  |  |  |
| Rate | 1,58 | 1,18 | 1,03 | 1,04 | 1,06 | 1,22 | 0,88 | 1,05 |
| 95% CI lower | 1,37 | 1,00 | 0,86 | 0,86 | 0,88 | 1,01 | 0,70 | 0,85 |
| 95% CI upper | 1,82 | 1,39 | 1,23 | 1,24 | 1,28 | 1,46 | 1,08 | 1,28 |
| PRETERM |  |  |  |  |  |  |  |  |
| Rate | 22,03 | 15,27 | 11,14 | 12,00 | 12,22 | 14,06 | 9,85 | 11,97 |
| 95% CI lower | 18,67 | 12,53 | 8,80 | 9,56 | 9,63 | 11,08 | 7,45 | 9,18 |
| 95% CI upper | 25,82 | 18,44 | 13,93 | 14,88 | 15,30 | 17,60 | 12,78 | 15,36 |
| TERM |  |  |  |  |  |  |  |  |
| Rate | 0,39 | 0,35 | 0,44 | 0,37 | 0,40 | 0,48 | 0,35 | 0,41 |
| 95% CI lower | 0,28 | 0,26 | 0,33 | 0,26 | 0,29 | 0,35 | 0,24 | 0,29 |
| 95% CI upper | 0,51 | 0,48 | 0,58 | 0,49 | 0,54 | 0,64 | 0,48 | 0,57 |
